# Elongation and branching of stem channels produced by positive streamers in long air gaps

**DOI:** 10.1038/s41598-021-83816-7

**Published:** 2021-02-18

**Authors:** Xiangen Zhao, Marley Becerra, Yongchao Yang, Junjia He

**Affiliations:** 1 State Key Laboratory of Advanced Electromagnetic Engineering and Technology, Huazhong University of Science and Technology, Wuhan, China; 2 School of Electrical Engineering and Computer Science, KTH Royal Institute of Technology, Stockholm, Sweden

**Keywords:** Natural hazards, Engineering, Physics

## Abstract

The elongation and branching of long positive spark discharges in the laboratory and in lightning have been attributed to the formation of thermalized channels inside a diffuse, glow-like streamer section at the leader head. It is experimentally shown here that the structured morphology of streamers produce low-density stem channels that elongate and branch well before a new leader channel section is formed. These non-thermalized stems are also shown to develop ahead of a developing leader channel. These findings are based on high-speed photography and Schlieren imaging used to visualize both the morphology of streamer filaments and stem channels. Numerical analysis is also performed to estimate the axial temperature and density of the stem channels. A stem-driven mechanism for the propagation and branching of positive long air gap discharges is proposed and discussed based on the presence of not-yet thermalized, low density channels formed by streamer ensembles at the leader head.

## Introduction

The knowledge of the physical mechanisms of long positive sparks is necessary to solve a variety of engineering problems and to understand natural lightning flashes^1–3^. Streamers and leaders are the two main mechanisms involved in the development of electrical discharges in long air gaps. Streamers are non-thermal plasmas composed by a large number of filaments, developing from a sharp electrode or at the tip of a thermalized leader channel. Mobile electrons in streamers converge in the positive leader and diverge in the negative leader^2,3^. These streamer filaments and the current associated to them converge into one or several roots known as stems^4,5^. As the current flows through to the narrow stem, the gas is heated locally above atmospheric temperature causing a decrease in gas density. Following the path of the lower-density stem channel, a leader discharge can later develop when the gas reaches a temperature threshold between 1500 and 2000 K^4,6^. At such a temperature, there is a sharp increase in the stem conductivity such that the transition from streamers into a thermalized leader channel occurs. Recent calculations have shown that a leader discharge can propagate into the gap only when the gas is heated well above 2000 K during the transition^7^.

Research on the development of streamer discharges in the laboratory and in natural lightning is rather abundant, both experimentally^8–13^ and theoretically^14–16^. However, the knowledge of their stems is still rather poor. Experimental data of the stem produced by positive streamers has been generally based on images obtained using standard photography, in which the bright region at the streamer root is regarded as the stem^17,18^. From these photographs, it is unfortunately difficult to observe the actual stem as it is outshined by the light emission from the streamer^19^. Only until recently, studies have started using Schlieren imaging^20,21^ to directly observe the low-density channels of stems and using computer simulation to understand in more detail their radial formation^22^. Unfortunately, no measurement or analysis is yet available about the elongation and branching of stems along the discharge axis.

Much experimental and theoretical research^5,23–28^ has also been carried out to characterize the axial propagation of positive leader discharges both in long air gaps and in natural lightning. Abundant streak and frame photographs available in the literature have indicated that leaders developed from a bright diffuse streamer region at the thermalized channel tip, known as the leader head. Based on these observations, it has been hypothesized that a leader channel propagates due to the energy provided by the streamer current converging into the head, when the temperature threshold is reached. Thus, positive leader discharges have been assumed to propagate continuously through short steps equal to the length of the bright diffuse zone observed at their heads^17^.

However, only recent high speed images of positive long air gap discharges have allowed the observation of the fine details of the propagation of the leader head^18^. They have clearly shown that the streamer filaments at the leader head do not converge into the bright diffuse zone observed in earlier studies using traditional photographs with long exposure times. Instead, streamers at the leader head have fine filamentary structures which can only be observed when short (lower than 2 μs) exposure times are used^18^. These finely structured streamer filaments converge into multiple bright roots branching from the leader channel tip. As the discharge proceeds, few of these branches can cause the spontanous bifurcation of the channel, which has been always observed with high speed photography in recent laboratory experiments^29,30^. Although branching is a very common feature observed in laboratory long air gaps and in lightning, the mechanism of positive leader bifurcation remains mysterious till now^31,32^.

In this paper, high speed Schlieren images of the low-density channels created by stems are complemented with traditional photographs detecting the streamer morphology. In the experiments, multiple separated streamer bursts are produced at the electrode tip. The Schlieren images show for the first time that stems created by streamer bursts elongate and branch, a process that is invisible in standard brightness photographs. The transient variation of the temperature and the density of the stems is estimated with a thermohydrodynamic model using the measured injected current as input. One event developing into a leader discharge is also analysed. Together with the observations and simulations, the stem at the leader head is compared with that at the electrode tip. At last, a mechanism of stem-driven propagation is proposed for positive leader discharges in long air gaps.

## Results

### General properties of streamers and stems

**Figure 1:**
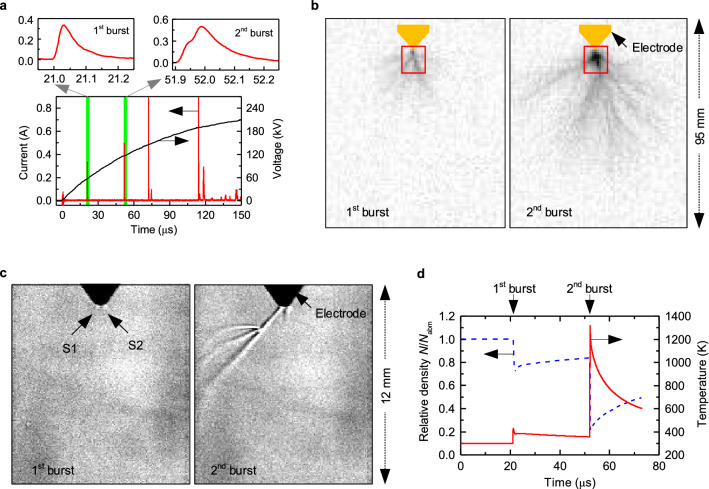
Typical results obtained in the experiment. (**a**) Current and voltage waveform, with the current pulses of the first two streamer bursts enlarged. (**b**) High-speed photograph frames of the morphology for the first and the second streamer bursts. The Schlieren observation area is indicated by the red box as reference. (**c**) High-speed Schlieren images of the stem channels formed by the first and the second streamer bursts. Images in (**b**) are colour inverted and those in (**c**) are contrast-enhanced. Frames between of these two bursts are displayed in the Supplementary Note 1. (**d**) Time variation of the stem density and temperature estimated from the measured current pulses.

Figure 1 shows typical recordings of the injected current as well as the brightness and Schlieren images of the first and second positive streamers measured during the risetime of a switching impulse voltage with a peak amplitude of 235 kV. Under the tested conditions, a sequence of multiple, separated streamer bursts are produced as shown by the current pulses in Fig. 1a. Note that the signal recorded at time zero is an electromagnetic disturbance due to the ignition of the Marx generator. As only the images during the first and the second streamer bursts are shown, only their corresponding current pulses are enlarged at the top of Fig. 1a. The first streamer current pulse has a risetime of 30 ns and a fall time of 210 ns, comparable with previous measurements^5^. After a dark period of 21 µs in this case, the second streamer current pulse has however a larger rise and fall time (of 89 and 243 ns respectively), as it is the result of the superposition of at least two asynchronous current pulses.

The streamer morphology of the first and the second bursts, obtained by integration of the emitted light (i.e. brightness) of streamer filaments during their development, is shown in Fig. 1b. The photographs are color-inverted for sake of visualization. All streamers appear to converge into a bright root observed as a darker area in front of the electrode tip. This root has been regarded in the existing research as the stem, where the localized heating takes place and a low-density gas channel is formed. Furthermore, the length of the stem channel is generally assumed equivalent to the axial extension of the observed bright root.

However, the observation of changes in temperature (or in gas density) rather than in brightness is the better way to truly visualize stem channels and to conclusively distinguish them from streamer filaments. This is here accomplished in the Schlieren images in Fig. 1c, showing only changes in the gas refractive index caused by density changes can be observed. For sake of visualization, the contrast of all Schlieren images is enhanced. In these images, the streamer filaments are not visible due to two reasons: (1) the streamer filament temperature is close to room temperature causing a minimal air density change; (2) the luminous intensity of the streamer filaments is much weaker compared with the auxiliary light source of the Schlieren system. Interestingly, two short stems (S1 and S2) can be recognised in the Schlieren images after the first streamer burst. Then, only one of them (S1) develops along its original direction during the second streamer burst. Surprisingly, this stem also branches at some distance from the electrode tip into two additional side channels. Comparison of Fig. 1b,c also shows that the bright region observed by standard photographs at the streamer root cannot be used alone to evaluate the number, axial length and morphology of the stems produced by the discharge, which are only clearly visible in the Schlieren images.

In order to estimate the density and temperature of the stem S1 in the Schlieren images in Fig. 1c, thermo-hydrodynamic simulations with a detailed chemical scheme for humid air are carried out as reported elsewhere^7,22^ using as input the measured current pulses of the first and second streamers in Fig. 1a. The first current pulse is divided by two as it is assumed that the current is equally shared by the two stems initially observed. Then, 90% of the second current pulse is assumed to flow through the dominant stem S1. The initial radius of the stem is estimated from Fig. 1c as 0.2 mm. Figure 1d shows the estimated time-varying density and temperature of the stem channel S1. Observe that the estimated temperatures show that the observed low-density channels correspond to stems since leader discharges would require higher temperatures above the thermalization threshold, here taken as 2000 K^7^. Thus, the stem temperature slightly increases to 400 K during the first streamer burst, while the ratio between the stem density *N* and the atmospheric density *N*_*amb*_ is about 0.7. This minor relative density decrease is visible by the faint changes in the refractive index at the stems S1 and S2 as observed in the Schlieren image of the first burst in Fig. 1c. During the dark period, the relative stem density *N*/*N*_*amb*_ slowly recovers to about 0.8 before suddenly dropping again at the inception of the second streamer burst to about 0.25. Furthermore, the stem reaches a temperature close to 1300 K at the current pulse maximum, which rapidly decreases due to strong convection losses produced by the gas expansion in the channel^7^. As a result, the streamer development is quenched followed by a second dark period.

### Stem elongation

**Figure 2:**
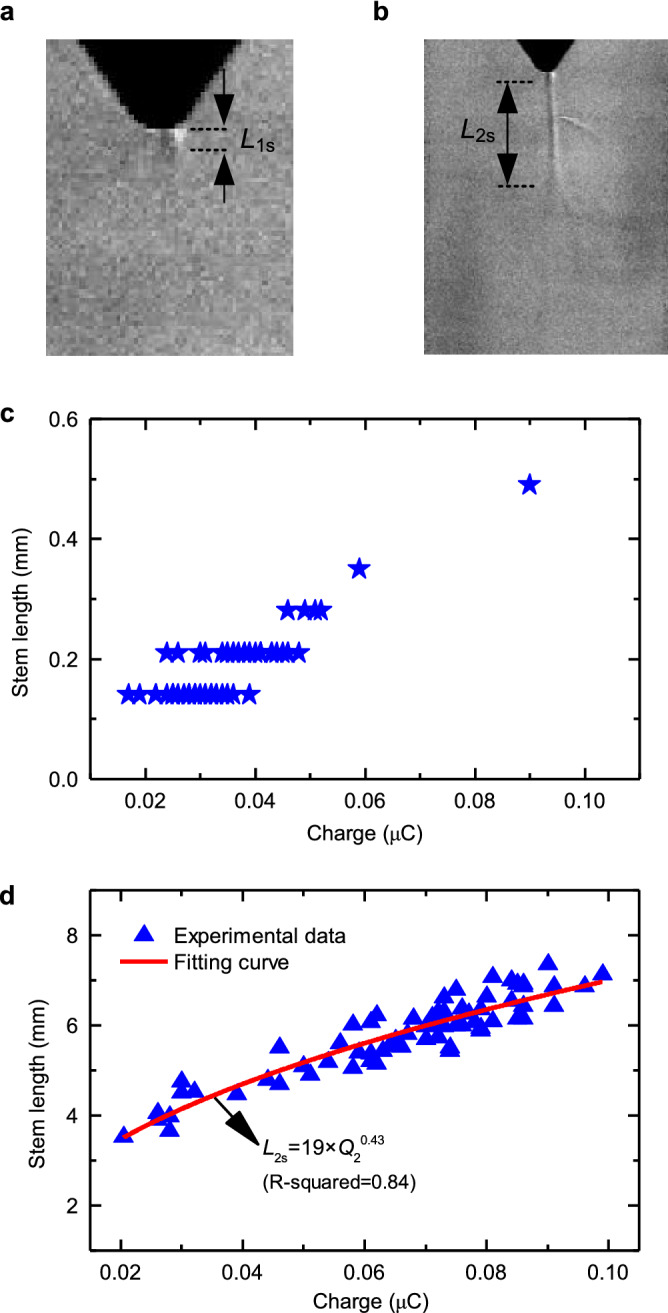
Stem elongation after the first and the second streamer bursts. Examples of the length measurement of stems produced by (**a**) the first burst and (**b**) the second burst. (**c**) and (**d**) show the measured stem length as a function of charge for the first and the second streamer bursts, respectively.

The length of the produced low-density channel is a key parameter to characterize the stem elongation. The method to measure this length is detailed in the Supplementary Note 2. Figure 2a,b shows two typical examples to illustrate the length measurement of stems produced by the first and second streamer bursts. Since the observed length is the integral effect of the changing current density flowing through the low-density channel, the total charge injected in each streamer burst is a more representative quantity to assess the stem development^22^. The estimation of streamer charge is shown in the Supplementary Note 3.

Figure 2c shows the estimated stem elongation as a function of the total charge for the first streamer bursts produced under switching impulse voltages with peak value ranging between 235 and 320 kV. The measured stem length varies from 0.14 to 0.49 mm for first streamer charges ranging between 0.01 and 0.09 μC. Note that the broad scatter observed in the data could be caused by the spatial resolution of the Schlieren system (70 μm/pixel) and by the formation of more stems than those observed. Due to this large scatter in the measured datapoints, no suitable analytical function relating these two variables can be found for the first streamer bursts. However, Fig. 2c shows that the charge per unit length required to elongate a single first streamer stem ranges between 110 and 280 μC/m with an average of about 200 μC/m. Observe that no elongation of the stem is observed during the dark periods as evident in the Supplementary Figures 1 and 2.

Compared with the first streamer, the stem associated to the second burst develops much longer under the same injected charge as shown in Fig. 2d. The stem length and streamer charge in the second burst vary from 3.53 to 7.35 mm and from 0.02 to 0.10 μC, respectively. The results clearly point out that the length of stems produced by positive streamers do not remain constant as generally assumed based on standard photographs. Instead, the stem length *L*_2s_ depends on the injected second streamer charge *Q*_2_, following a relationship that can be best-fitted to a power law function as:

1 $${L}_{2s}=19\times {Q}_{2}^{0.43}$$

where the units for *L*_2s_ and *Q*_2_ are mm and μC, respectively. From Fig. 2d, the charge per unit length required to elongate a single stem produced by a second streamer burst is estimated to range from 5 to 15 μC/m, with an average of about 11 μC/m.

### Stem branching

**Figure 3:**
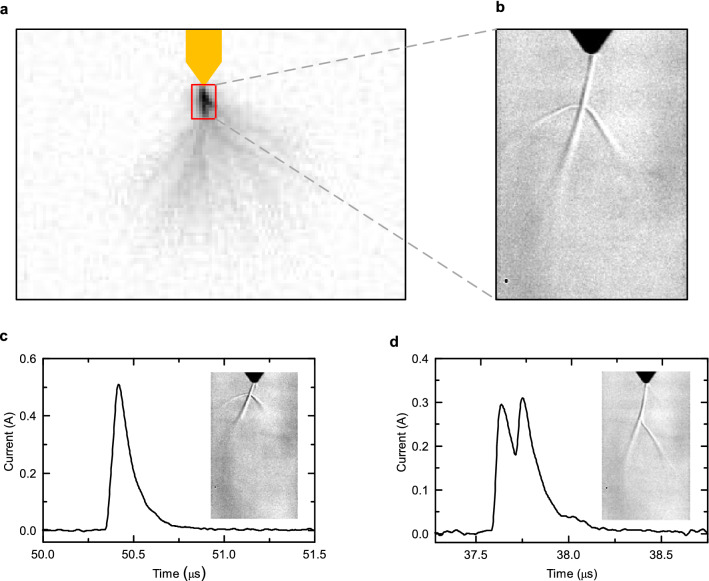
Branching of stems produced by second streamer bursts. (**a**) High-speed photograph of a streamer burst with two distinct filament ensembles. (**b**) Corresponding Schlieren image of the low-density channels branching from a main stem channel. (**c**) and (**d**) show two events with different types of current waveforms measured during stem branching.

Figure 3a,b shows the typical branching morphology of the second streamer burst and of the formed stem. In this case, the traditional photograph shows that this second streamer is formed by a dominant, forward filament ensemble with one distinct, side filament ensemble merging at a distance from the electrode tip. Although additional off-electrode filament ensembles could have been present in this event, they are not detected if located outside the focal length of the optical system. The complementary Schlieren image shows that a main stem accompanied with two side branches is formed in this streamer burst. Comparison between the brightness and Schlieren images conclusively allow to correlate the branching in the stems with the locations where different streamer filament ensembles merge. Consequently, the current collected at the root of off-electrode filament ensembles locally heats the gas and creates a low-density branching channel connected to a main stem. The location and number of branches in the stems observed throughout the experiment have been found to vary randomly following the stochastic process of streamer branching.

In order to assess whether the stem branches are formed simultaneously, the streamer current waveforms are analyzed for two additional second streamer events in Fig. 3c,d. It is found that there are events in which the stem branches are formed simultaneously as the measured current waveform, collecting the synchronous current of each streamer filament ensemble, is a single, double exponential pulse. However, other events show a clear small-time shift (of 114 ns in Fig. 3d) between the current of two different filament ensembles, resulting into a measured current pulse with two distinct peaks. No consistent trend for the synchronous or asynchronous formation of stem branches could be identified as they these events appear randomly in the experiments.

### Effect of stem elongation and branching on following leader development

**Figure 4:**
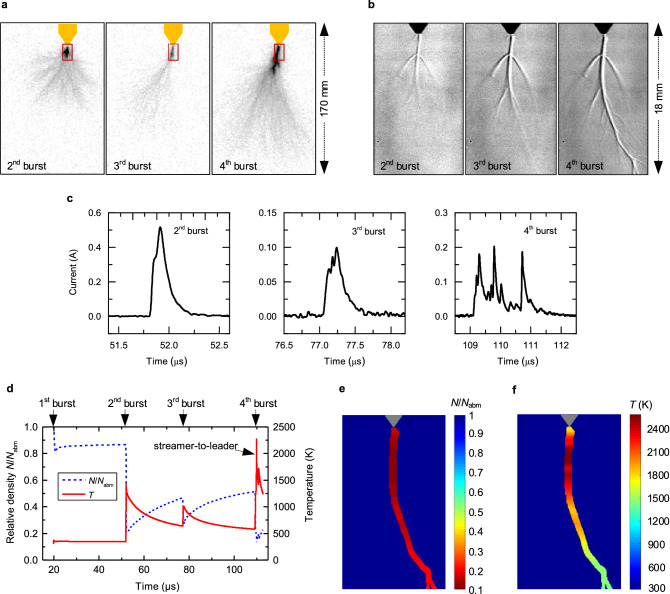
Experimental and simulated results to show the effect of stem development on the propagation and branching of long positive sparks. (**a**,**b**) High-speed frames of streamer morphology and Schlieren images of low-density channels for the second, third and fourth streamer bursts. The Schlieren observation area is indicated by the red box as reference in (**a**). Frames between of these bursts are displayed in the Supplementary Note 1. (**c**) Current pulses for the mentioned streamer bursts. (**d**) Simulated time variation of the temperature and density at the channel base on the electrode. (**e**,**f**) Estimated spatial maps of the minimum relative density and maximum temperature along the main channel after the streamer-to-leader transition during the fourth burst.

As the applied voltage continues increasing after the second streamer, the discharge can further develop. Figure 4 shows images, current waveforms and estimated density and temperature of an event in which two additional streamers are produced after the second burst, one of them developing into a leader discharge. In this case, the elongated and branched stem channel produced by the second streamer leaves a track of low relative density *N*/*N*_amb_ (estimated to be about 22% at the stem base at 51.7 μs in Fig. 4d). After an additional dark period, the density of the stem channel recovers but still remains low (at about 0.45 *N*_amb_ at 77 μs), defining a narrow path with intensified density-normalized electric field *E*/*N* (compared with the surroundings) at the initiation of the third burst. Then, the root of this third discharge develops along that preferred path, defining a narrower streamer morphology along the extension of the stem produced by the second burst as observed in Fig. 4a. As the streamer continues developing beyond the existing stem into the gap, it branches into a large number of filament ensembles. This new streamer ensembles inject an additional current pulse which forms a new branched stem section as shown in Fig. 4b. Then, this current concentrates on the narrow crosssection of the existing stem and flows into the electrode. As a result, the stem base temperature is rapidly increased to only about 1000 K, such that the discharge cannot continue and an additional dark period occurs.

Under the increasing applied voltage, the stem produced by the third streamer also leaves a low-density track with intensified *E*/*N* along which the fourth discharge develops in a similar manner. Thus, the incepted fourth streamer is narrow at its root and branches into the gap ahead of the path of the remaining stem. The injected current pulse at the inception of this fourth streamer is concentrated in the stem cross section, further heating the channel base to about 2000 K with the resulting drop in gas density (at 109.3 μs in Fig. 4d). At such high temperature, the stem base is thermalized and transformed into a leader channel. This streamer to leader transition allows the development of additional streamer ensembles which produce additional, delayed superimposed current pulses as shown in Fig. 4c. However, the temperature of this channel as shown in Fig. 4d cannot be maintained high enough to sustain the further development of the leader due to the strong convection losses around this thermalized channel^22^. For this reason, no additional streamer bursts can be generated after few microseconds and the leader discharge is aborted.

Analysis of both the Schlieren image measured during the fourth burst and the injected current allows to estimate the density and temperature spatial maps along the main low-density channel after the streamer-to-leader transition. Figure 4e shows that the main channel observed in the Schlieren image during the transition have density significantly lower the surroundings. While the channel base on the electrode reaches a relative density of about 0.13, the faintier newly formed channels reach an estimated *N*/*N*_*amb*_ of 0.23. More significant differences can be found in the estimated temperature of the channels in Fig. 4f. Observe that the temperature along the entire extension of the main stem left by the previous bursts is above 2000 K after the transition, as the result of the concentration of the fourth current burst in the small radius of the pre-existing low-density channel. At such a temperature, additional ionization processes increase the lifetime of the plasma^6^ such that the entire existing stem is thermalized into a leader channel with low electric field. Interestingly, the temperature of the new channels created by the branched streamer ensembles ahead of the newly-formed leader channel, gradually decrease to values as low as 1300 K. Thus, these new low-density channels connected to the leader channel remain non-thermalized, having similar properties as the stems connected to the electrode during the streamer bursts.

## Discussion

The streamer stem has traditionally been considered as a low density region of negligible length in front of the electrode, which is only relevant for the inception of positive leader discharges^5^. Within this context, the streamer stem has been generally treated as a cylindrical plasma channel in front of the electrode^5,33^, which is heated up until the first leader channel is initiated. The Schlieren images here reported have however shown that stems are not just passive regions at the root of streamers initiated from the electrode. They also elongate and branch, features which were not known until now due to the limitations of standard photography. Note that the stem elongation could be in continuous or stepping mode. However, the results above correspond to the latter mode as a consequence of the pulsed streamers observed in the experiments.

The elongation of stems during streamer bursts have been here correlated to the injected charge, as it is generally assumed for leader discharges^1^. The first streamer generally produce very short stems (a fraction of a millimeter long) with density slightly lower than atmospheric, requiring a significant amount of charge (up to 220 μC/m) to elongate the stem channel per unit length. In turn, the current injected by second streamers causes a significant decrease of the density of stem channels (below 30% of atmospheric conditions), making them elongate further (several millimeters long) with a significantly lower charge per unit length (up to 15 μC/m) compared with the first streamer. Since more stems than those observed in the Schlieren images could have been produced in the experiment^21,34^, the values of charge required for the elongation of a single stem per unit length here reported should be however treated as upper-bound estimates. Despite of this uncertainty, the reported upper-bound charge per unit length required for a unit length elongation of the stem after the second streamer is comparable with the corresponding value reported for the propagation of leader channels in the laboratory, ranging between 18 and 50 μC/m^5^.

The formation of stem branches along a main stem channel observed in the Schlieren images is correlated to the formation of streamer ensembles having an off-electrode root as observed by standard photography. This may be related to the streamer discharge structure named the collective streamer front^35^. After the streamer firstly bifurcates, positive charges at each newly formed filament tip enhance the field ahead and facilitate the emergence of more streamer branches. As a result, the initial streamer branch becomes the root of the subsequent streamer branches. The electric current produced by the subsequent branches contributes to the heating process of its root, and a stem channel can form.

The formation, elongation and branching of new stem channels away from the electrode have been here reported for the first time. This process has been observed at the tip of a newly formed thermalized channel, as observed in the temperature map estimated during a streamer to leader transition event. These elongating and branching stems can transform into thermalized channels if sufficient electrostatic energy is available for further development of streamer ensembles from them. Thus, it is here clearly shown that streamer stems are not only important for the inception of the leader channel. Elongating and ramifying stems will also affect the propagation and the branching of leader discharges, as stems are also produced by streamer ensembles at the head of a thermalized channel. Although this process is here observed during the early stages of an aborted leader, recent photographs captured with short exposure times have shown that streamer ensembles with different roots also converge into the tip of a continuously propagating leader^18^. Interestingly, these filament ensembles look similar to those for streamer bursts from sharp electrodes as shown here. Thus, the presence of stems at the tip of a leader channel is a likely process which challenges the traditional view of the thermalized channel head as a bright and diffuse, glow-like region^5,36^.

The traditional view of diffuse streamer ensembles starting from the leader tip however requires a mechanism for the current of multiple filaments to concentrate locally in order to enable thermalization within a region of small radius at the head^36^. While this mechanism has been completely ignored in early studies^5^, it has been later attributed to the current constriction caused by an ionization-thermal instability in the leader head, in a similar way as observed in glow discharges^17^. Despite of several subsequent studies adressing such a mechanism^6,17,37^, the problem of the evolution of a positive leader channel has not yet been completely solved.

Since low-density stems are also present at the leader head as reported here, an alternative mechanism to those suggested by Gallimberti^5^ and Bazelyan and Raizer^36^ is also proposed. Instead of assuming that the streamer-to-leader transition in front of an electrode is entirely different to that at the head of a thermalized channel, it is here suggested that stems are also produced during the positive leader development. These non-thermalized stem channels are formed by localized concentration of the current at the root of one or several streamer ensembles formed from the leader tip. This stem formation occurs in the same way as for pulsed subsequent streamers initiated from the tip of the electrode (e.g. Fig. 3). In the presence of these low-density stems, some of which could be branched, one or more preferred paths with intensified *E*/*N* are available for the further development of the discharge. In this manner, an efficient concentration of the current produced by streamer ensembles at the leader tip is produced, which further heats the stem(s). Under normal conditions, one single stem concentrating the current of the dominant streamer ensemble will eventually be thermalized and a new leader segment will be formed from it. Depending on the spatial electrostatic energy distribution in the gap, two streamer ensembles connected to a branched stem at the leader tip could also dominate, such that two new low-density sections are thermalized instead and a leader branch is observed. This branching process will continue with the further development of two different leader heads with their corresponding streamer ensembles if enough electrostatic energy is available, causing a sudden increase in the injected current as observed in experiments^29^. The formation of a leader branch will therefore be influenced by the stochastic nature of the streamer stem formation and by the available three-dimensional spatial distribution of the electrostatic energy in the gap.

Thus, the propagation of a leader discharge in long air gaps is proposed to be a process driven by the elongation and branching of stems ahead of the already thermalized channel. Although the pulsed streamers in front of the electrode here reported include a dark period, which is not present for streamers at the tip of a leader, both are generally considered to be analog^37^. Observe that the leader channel is always regarded as the elongation of the high-voltage electrode into the gap and the electric field around the leader tip is similar to that around the electrode tip. Furthermore, there is solid experimental evidence that the propagation of positive leaders taking place is small steps^17^, causing sudden transient changes in the tip electric field. Furthermore, the short dark period here observed (of less than 30 µs) also guarantees that the positive ion migration between streamer bursts is almost negligible as for streamers at the leader tip. The ions move a distance of less than 2 mm with an average speed of 100 m/s^38^, which is much shorter than the streamer length (25 mm in Fig. 1b). Similarly, most residual vibrational states produced by the streamer bursts, which also contribute to gas heating, remained during the dark periods in the experiment as the time scale of the vibration-to-translation (VT) relaxation is around tens of milliseconds^7^. Thus, the stems produced by streamers bursts as those here reported can be considered to be similar to those produced by streamer ensembles at the leader tip.

The new positive leader propagation mechanism that is here suggested does not only have just implications for long air discharges in the laboratory. As the streamers optically observed at the heads of natural positive leaders^11–13^ show some similarities with those in the laboratory discharge, the proposed mechanism also introduces a new physical process which needs to be assessed when studying leader-related lightning phenomena such as intermittent propagation of positive leader^39–44^, recoil leaders^45–47^ and needles^12,48,49^. According to this new mechanism, the leader will propagate stepwise using short steps, as previously suggested by Bazelyan^17^. However, the length of the step is not given by the optical length of the bright region at the head observed by standard photography. Instead, the steps are given by the rate of thermalization of one or two stems formed at the head. Similarly, the proposed mechanism also implies that low density stem branches are left all along a leader channel. These low-density branched channels define pre-existing tracks of high density-normalized field *E*/*N* along which the discharge can further rapidly develop, reilluminate and probably emit intense high-frequency in response to sudden changes in the spatial electrostatic field distribution caused by the tortuous propagation of the lightning channel. However, one major challenge to further understand the stem-driven propagation of leader discharges is the existing lack of knowledge about the streamer root and the associated formation of stems. Thus, there is a need for further theoretical and experimental research to fully understand and quantify the mechanisms of streamer branching and root formation as well as the process of inception and axial development of low-density stem channels.

## Methods

### Experimental setup

**Figure 5:**
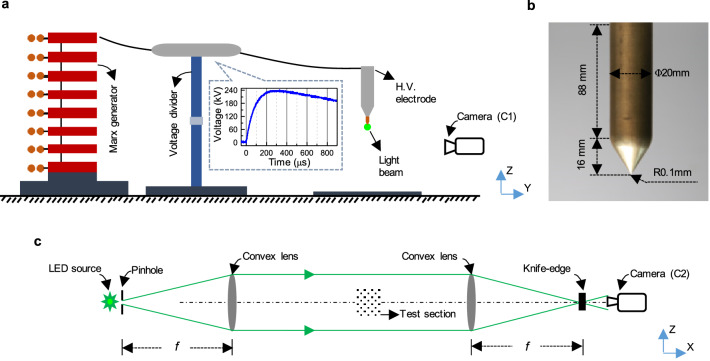
Experimental setup for multiple streamer bursts including Schlieren photography (not to scale). (**a**) Schematic of the experimental setup. The high-speed camera (C1) is used to observe the streamer morphology. The green circle at the electrode tip marks the light beam of the Schlieren system. (**b**) Details of the cone-shaped brass electrode. (**c**) Diagram of the Toepler’s lens-type Schlieren imaging system to the second high-speed camera C2.

The sketch of the experimental setup is shown in Fig. 5a. A Marx generator is used to produce positive switching impulse voltages of 300/2000 μs rise/fall time. The gap distance used in our experiments is 1.4 m to the ground plane. Peak voltages of 235, 295 and 320 kV are applied for the production of multiple separated streamer bursts without breakdown. A sharp cone-shaped electrode, which is detailed in Fig. 5b, is installed as high voltage terminal to minimize the number of separate stems formed for each streamer burst. Even though the designed value for the tip radius is 0.1 mm, the electrode becomes blunter after several experiments.

The impulse voltage is recorded by a Tektronix DPO-4104B digital oscilloscope together with a capacitor divider (ratio: 1:759). A shunt resistor of 25 Ω is used to measure the streamer current at the sharp electrode. The measured analog signal is locally recorded by a data acquisition card with a sampling rate of 500 M samples per second. In order to reduce the electromagnetic interference and to ensure the electrical safety, the current measurements are locally converted into optical signals and then transmitted. All experimental data are synchronized with the same method described elsewhere^34,50^. The experiments are performed at a temperature of 307 K, absolute humidity of 23.4 g/m^3^ and atmospheric pressure.

### Standard photography and Schlieren imaging systems

A high-speed camera (C1: Phantom V1212) equipped with a Nikon 50 mm F1.4 fixed lens is employed to observe the streamer morphology. This camera C1 is placed 2 m away from the electrode and is directed perpendicular to the air gap axis. The camera C1 is operated at a maximum frequency of 190,000 frames per second (fps) with an exposure time of 3.72 μs, using a spatial resolution of 128 × 256 pixels. The corresponding observation area is 180 × 360 mm^2^.

The low-density channels in the discharge are visualized with a Toepler’s lens-type Schlieren imaging system. It consists of two convex lenses, a light source, a pinhole, a knife-edge and a high-speed camera (C2: Photron FASTCAM SA-X2), as illustrated in Fig. 5c. Both convex lenses have a focal length (*f*) of 1500 mm and an aperture of 150 mm. A pinhole with a diameter of 6 mm and a knife-edge are located at the focal points of the convex lenses. The direction of the knife-edge is vertical, i.e., parallel to the air gap axis. The system is illuminated by a 50 W light-emitting diode (LED) with a central wavelength of 512 nm. In order to obtain high spatial and temporal resolutions, the camera C2 is equipped with a Sigma 300–800 mm F/5.6 zoom lens and is operated at 200,000 fps with an exposure time of 3.98 μs. A frame size of 152 × 256 pixels is used with a corresponding spatial resolution of 70 μm.

### Numerical analysis

The transient variation of the axial temperature and density of the stems is obtained with the one-dimensional thermal-hydrodynamic model developed and described in detail elsewhere^7,22^. It includes an extensive chemical scheme with 45 species and 192 chemical reactions for humid air at the conditions of the experiment. The model contains a set of continuity equations for the number density ${n}_{i}$ of the *i*th specie considered (2), the transport of momentum $\rho v$ (3) as well as the conservation equations for the overall translational energy density $\varepsilon$ (4) and the vibrational energy density of ${\mathrm{N}}_{2}$ molecules ${\varepsilon }_{\mathrm{V}}$ (5) as follows:

2 $$\frac{\partial {n}_{i}}{\partial t}+\nabla \cdot \left[{n}_{i}{u}_{i}-{D}_{i}\nabla {n}_{i}\right]={S}_{i}$$

3 $$\frac{\partial \rho v}{\partial t}+\nabla \cdot \left(\rho vv\right)=-\nabla P+\frac{1}{3}{\mu }_{v}\nabla \left(\nabla \cdot v\right)+{\mu }_{v}{\nabla }^{2}v$$

4 $$\frac{\partial \varepsilon }{\partial t}+\nabla \cdot \left[\left(\varepsilon +P\right)v\right]={Q}_{\mathrm{T}}^{\mathrm{eff}}+\nabla \cdot ({\kappa }_{T}^{*}\nabla {T}_{g})$$

5 $$\frac{\partial {\varepsilon }_{\mathrm{V}}}{\partial t}+\nabla \cdot \left({\varepsilon }_{\mathrm{V}}v\right)={Q}_{\mathrm{V}}^{\mathrm{eff}}+\nabla \cdot ({D}_{V}\nabla {\varepsilon }_{\mathrm{V}})$$

where ${u}_{i}$, ${D}_{i}$ and ${S}_{i}$ denote the number density, drift velocity, diffusion coefficient, and net reaction rate respectively. $\rho$, $v$, $P$, and ${\mu }_{v}$ are mass density, bulk velocity, gas pressure, and viscosity coefficient, respectively. The term ${Q}_{\mathrm{T}}^{\mathrm{eff}}$ accounts for the effective rate of energy deposition in the translational degree of freedom, ${\kappa }_{\mathrm{T}}^{*}$ and ${T}_{g}$ being the thermal conductivity and the gas temperature, respectively. ${Q}_{\mathrm{V}}^{\mathrm{eff}}$ accounts for the effective rate of energy deposition in the vibrational degree of freedom and ${D}_{V}$ the diffusion coefficient of ${\varepsilon }_{\mathrm{V}}$. The input to the above model is the measured current waveform, and the initial thermal radius of the channel for the model is estimated from the Schlieren images.

The maximum temperature and minimum density maps for the event in Fig. 4 are obtained by first estimating the relative temperature in the channels from the Schlieren images. The relative variation of temperature along the low-density channels is estimated with an Abel inverse integral transform as detailed in our previous work^50^. Eighteen points along the main low-density channel are selected to calculate the average relative temperature. The corresponding values all along the channel are linearly interpolated. Since most parts of the main channel (in Fig. 4f) are not perpendicular to the air gap axis, one step in the process should be changed. Thus, the image intensity variation is multiplied by the cosine of the deflection angle due to two facts: (1) the channel can be approximated as deflection only in a certain plane as the channel in the red boxes in Fig. 4a is nearly vertical and (2) the temperature gradient in the axial direction can be ignored along a short channel section (less than 1 mm here). The temperature and relative density along all side branch channels are not shown as it is difficult to estimate their true deflection direction from the two-dimensional images in Fig. 4a,b. Then, the absolute temperature distribution along the channel is obtained by normalizing the relative temperature map to the axial temperature at the stem base on the electrode, obtained with the thermo-hydrodynamic model. Since the channel in the discharge becomes isobaric soon after the streamer initiation^7^, the density map is calculated from the temperature map by using the gas law at atmospheric pressure.

## Supplementary Information

Supplementary Information.

## Data Availability

All the raw and derived data that support the plots whin this paper and other findings of this study are available from the corresponding authors upon request.
