# Supplementary material for: Elongation and branching of stem channels produced by positive streamers in long air gaps
Source: Sci Rep. 2021 Feb 18;11:4120. doi: 10.1038/s41598-021-83816-7 (PMC7892885; doi:10.1038/s41598-021-83816-7)
Supplement: Supplementary file 1 — Supplementary Information. [file 41598_2021_83816_MOESM1_ESM.pdf]

Supplementary Information of

Elongation and Branching of Stem Channels Produced by Positive Streamers in Long Air Gaps

Xiangen Zhao[1*], Marley Becerra[2*], Yongchao Yang[1] & Junjia He[1]

[1] State Key Laboratory of Advanced Electromagnetic Engineering and Technology, Huazhong University of Science and Technology, Wuhan, China

[2] School of Electrical Engineering and Computer Science, KTH Royal Institute of Technology, Stockholm, Sweden

Supplementary Note 1: Detailed frames between the bursts in Figures 1 and 4

Supplementary Figures 1 and 2 shows all the frames between the bursts in Figures 1 and 4, respectively, in which the traditional photographs are also color-inverted for sake of visualization, and the Schlieren images are enhanced.

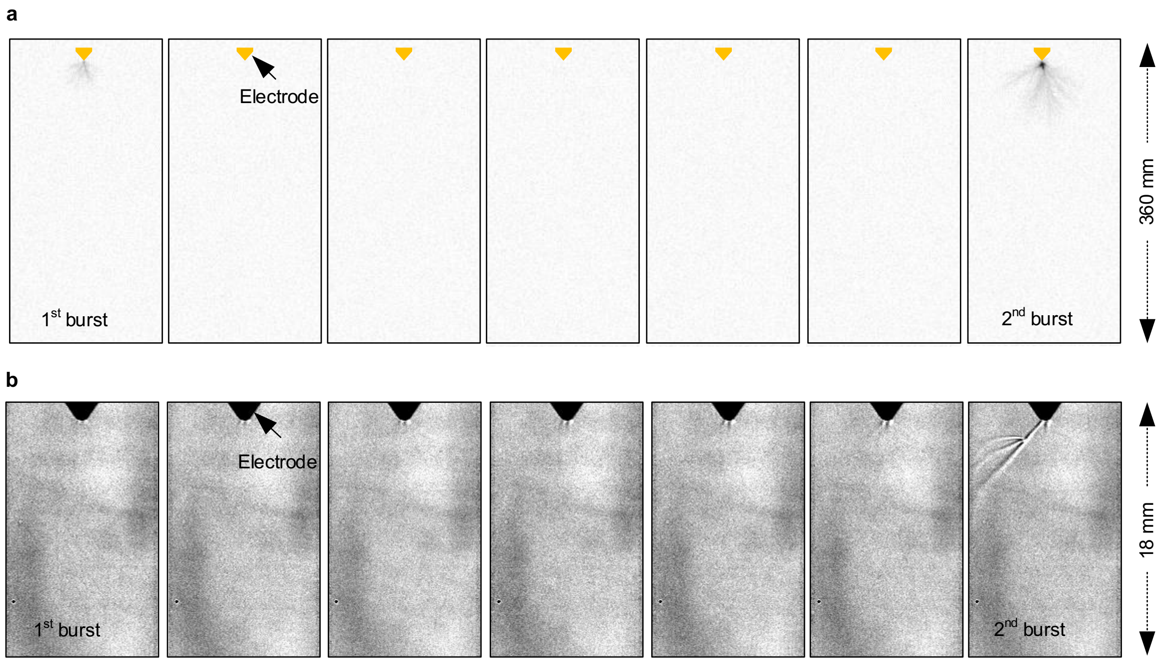

Supplementary Figure 1: All frames between the first and second bursts in Fig. 1. **a** High-speed traditional photographs and **b** High-speed Schlieren images.

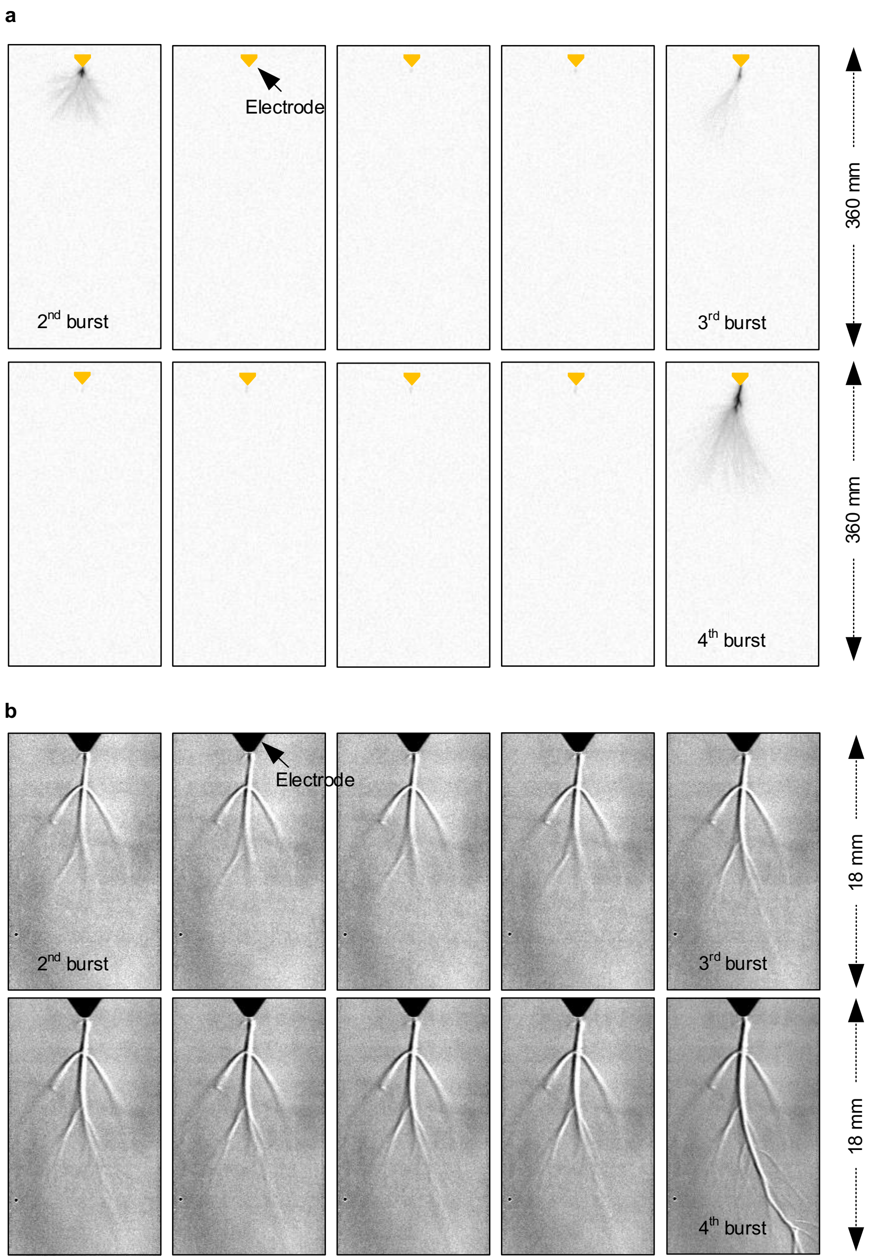

Supplementary Figure 2: All frames between the second, third and fourth bursts in Fig. 4. **a** High-speed traditional photographs and **b** High-speed Schlieren images.

Supplementary Note 2: Method to locate the starting and ending points of a stem

The key point to measure the length of a stem is to determine its starting point (Ps) and ending point (Pe), as shown in Supplementary Figures 3a and 3b. For the first corona, only the stem that grows in the second burst is measured. The starting point for the first stem is located at the connecting point with the electrode. For the second streamer, the starting point is located at the end of the first stem along which the new channel always continues to grow. For both the first and the second streamer stems, the ending points are defined at the cross section where the difference ΔG_{max} (as shown in Supplementary Figure 3c) between the maximum and minimum image intensity variations starts to be less than 25. The image intensity variation ΔG can be obtained by subtracting the gray value of the Schlieren images before the test from that with the discharge channel. Then the stem length is obtained as the distance between Pe and Ps.

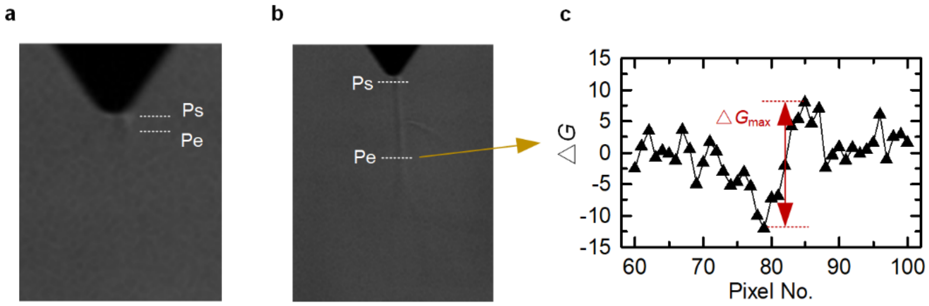

Supplementary Figure 3: Example of the stem length measurement. **a** and **b** Illustrations for the starting and ending points of the first and second streamer stems in the raw Schlieren images. **c** Image intensity variations along the cross section at the ending points in Supplementary Figure 1b.

Supplementary Note 3: Charge estimations for stem elongation

The charge for each streamer corona is calculated by time integrating the corresponding current pulse in this paper. Considering the existence of stem branches, there are two points to note in the charge calculation. First, if several distinct branches are formed (for instance in Supplementary Figures 4a Fig. 4b), the charge for each stem branch is calculated by dividing the total streamer charge by the number of stems. Second, if there is a clearly dominant main stem (Supplementary Fig. 4c) or if only small branched stems are present along the main channel (Supplementary Fig. 4d), the total charge is considered to contribute only to the elongation of the main stem.

a b c d

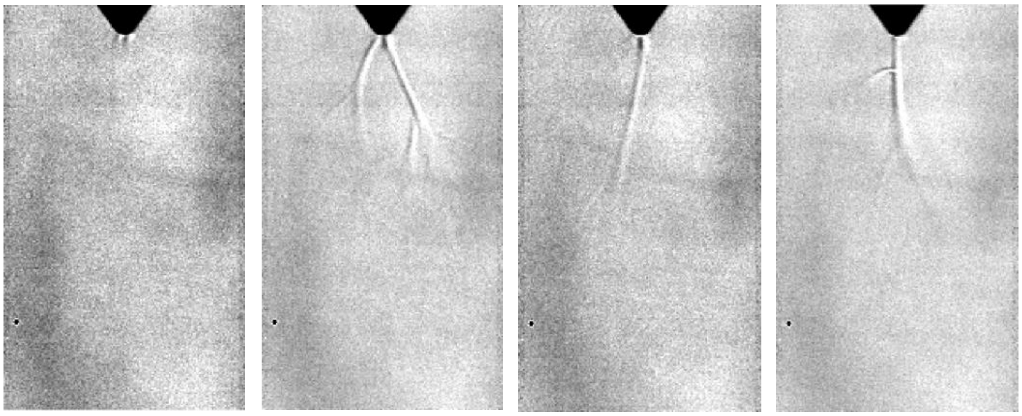

Supplementary Figure 4: Typical Schlieren images. **a** and **b** show cases that the streamer charge is considered to be equally shared by two stems. **c** and **d** Cases that the entire charge is considered to flow through the main stem.
